# Effects of Surface-Etching Systems on the Shear Bond Strength of Dual-Polymerized Resin Cement and Zirconia

**DOI:** 10.3390/ma17133096

**Published:** 2024-06-24

**Authors:** Sang-Hyun Kim, Kyung Chul Oh, Hong-Seok Moon

**Affiliations:** Department of Prosthodontics, Yonsei University College of Dentistry, 50-1 Yonsei-ro, Seodaemun-gu, Seoul 03722, Republic of Korea; andrew_krkr@naver.com (S.-H.K.); kyungabc@yuhs.ac (K.C.O.)

**Keywords:** zirconia, shear bond strength, acid etching, thermocycling, ceramic bonding, surface conditioning, cementation

## Abstract

Adhesion of zirconia is difficult; thus, etching agents using several different methods are being developed. We investigated the effects of surface treatment with commercially available etching agents on the bond strength between zirconia and resin cement and compared them with those achieved using air abrasion alone. We used 100 zirconia blocks, of which 20 blocks remained untreated, 20 blocks were sandblasted, and 60 blocks were acid-etched using three different zirconia-etching systems: Zircos-E etching (strong-acid etching), smart etching (acid etching after air abrasion), and cloud etching (acid etching under a hot stream). Each group was subjected to a bonding procedure with dual-polymerized resin cement, and then 50 specimens were thermocycled. The shear bond strengths between the resin cement and zirconia before and after the thermocycling were evaluated. We observed that in the groups that did not undergo thermocycling, specimens surface-treated with solution did not show a significant increase in shear bond strength compared to the sandblasted specimens (*p* > 0.05). Among the thermocycled groups, the smart-etched specimens showed the highest shear bond strength. In the short term, various etching agents did not show a significant increase in bond strength compared to sandblasting alone, but in the long term, smart etching showed stability in bond strength (*p* < 0.05).

## 1. Introduction

Over the past few decades, dentists have seen an increase in the demand for aesthetic restorations. Zirconia is extensively adopted for such restorations [1]. However, pretreatment is required for the clinical use of this material. Pure zirconia undergoes phase shifts with an increase in temperature, changing from a cubic phase to a monoclinic phase as it cools after sintering [2]. The addition of stabilizing oxides such as CaO, MgO, CeO_2_, and Y_2_O_3_ to pure zirconia enables the formation of a multiphase material known as partially stabilized zirconia [3]. Among these, the addition of 2–3 mol% Y_2_O_3_ stabilizes the tetragonal phase at room temperature, enhancing the properties of zirconia and forming yttria-stabilized tetragonal zirconia polycrystals (Y-TZP). As a tetragonal phase, Y-TZP is stable at room temperature, and its physical properties, including its fracture toughness and strength, are superior to those of alumina [4].

Generally, hydrofluoric etching and silanization are used as pretreatment techniques to bond resin to conventional dental ceramic restorations [5,6]; however, they do not increase the strength of the bond between the resin and the alumina or zirconia [7]. Zirconia bonding is the most difficult type of ceramic bonding, and bond failure is common during clinical trials [8]. According to a study on zirconia adhesion, when resin cements were used without an adhesive monomer, a combination of air abrasion and priming was needed to achieve durable long-term bonding to zirconia [9,10,11,12,13,14,15,16]. In addition, this treatment resulted in a high bond strength; several researchers have reported similar results [17,18,19,20]. Accordingly, several clinicians have performed zirconia adhesion using an adhesive resin cement containing 10-methacryloyloxydecyl dihydrogen phosphate after performing air abrasion during zirconia bonding.

Zirconia is subjected to air abrasion as a pretreatment to increase its bond strength with resin cement. In principle, air abrasion cleans the surface, removes impurities, increases the roughness of the surface, and changes the surface energy and wettability [21,22,23,24,25,26,27,28]. Furthermore, the silica nanoparticles that are emitted during the process not only loosely cover the abraded ceramic surface after the abrasion process but also cause the release of kinetic energy in the form of thermal energy, which results in the melting of the ceramic surface and the formation of zirconium silicate [29]. From this perspective, changes to the zirconia surface, such as roughening and phase changes, are crucial factors to consider.

In implant dentistry, hybrid zirconia abutments are often used for anterior implant prostheses that require superior aesthetics [8]. To complete prosthesis implantation with a hybrid zirconia abutment, adhesive cement is applied at two interfaces: between the titanium base and zirconia abutment and between the zirconia abutment and zirconia crown. The strong adhesion of zirconia is crucial to avoid adhesive failure at the interface between zirconia and cement [9].

According to a previous study [19] on the effect of surface treatment with zirconia based on air abrasion, several companies have proposed etching agents that chemically treat the zirconia surface to increase the bond strength with resin cement. However, these manufacturers claim that these chemical surface treatments can effectively increase adhesive strength on the basis of their own experimental data, and independent experimental studies on such effects have not yet been reported. Therefore, the aim of the present study was to investigate whether surface treatments with the etching agents proposed by various companies increase the bond strength between zirconia and resin cement compared to that attained with air abrasion alone. The null hypothesis is that pretreatment of zirconia with commercial etching agents would result in a higher adhesion strength than that attained with air abrasion alone.

## 2. Materials and Methods

One hundred cubic blocks (12 mm × 12 mm × 12 mm) were milled from 3Y-TZP (Plus Zir Block; DMAX Co., Ltd., Daegu, Republic of Korea) using a milling machine (MAXX-5Z; Robots and Design, Pangyo, Republic of Korea) and computer-aided manufacturing software (GO2cam Dental V6.09; GO2cam Intl., Lyon, France) (overlap tool diameter: 0.0750%; overlap volume: 0.1500; XY scallop: 0.0028). The blocks were sintered at 1500 °C for 7 h in a sintering furnace (Sintramat; Ivoclar Vivadent AG, Schaan, Liechtenstein) and randomly assigned to five groups; the random surfaces of the blocks in each group were subjected to a different surface treatment before the bonding procedure. Each group was further divided into two subgroups based on whether thermocycling was performed (*n* = 10 per subgroup). In naming the specimens, these subgroups were distinguished using “T” for the specimens that underwent thermocycling and “N” for those that did not. All milled surfaces were polished with 1500-grit sandpaper for standardization before testing.

The control-group blocks (groups N-P and T-P) did not undergo additional mechanical surface treatment after sintering. The blocks from groups N-S and T-S were sandblasted with Al_2_O_3_ particles using a sandblasting unit (Basic Master; Renfert GmbH, Hilzingen, Germany). The blocks from groups N-Z and T-Z were acid-etched for 2 h with Zircos-E etching solution (Zircos-E; Bioden, Seoul, Republic of Korea). The blocks from groups N-M and T-M were sandblasted for 15 s and then acid-etched using a smart-etching solution (Smart-etching; YesBio, Seoul, Republic of Korea) for 10 min in a hot water bath. Lastly, the blocks from groups N-C and T-C were acid-etched for 10 min in a hot steam pot (Cloud system; MEDIFIVE Co., Incheon, Republic of Korea) (Table 1). The properties of the materials used in this study are presented in Table 2.

Each group of blocks underwent the same bonding process after surface treatment. The surface-treated zirconia blocks were ultrasonically cleaned for 60 s and rinsed thoroughly with water. Subsequently, a primer (ED primer; Kuraray Noritake, Niigata, Japan) was applied to the blocks. Resin cement was applied to half of a size 5 gelatin capsule (PureCaps USA, Sudbury, MA, USA), which was then placed on the zirconia surface (bonding area: 12.56 mm^2^). Photopolymerization was performed using a 1000-mW light curing machine (LED.B; Guilin Woodpecker Medical Instrument Co., Ltd., Guilin, China) while the specimens were pressed using a 1-N weight. The resin-bonded zirconia specimens were then stored in water at 37 °C for 24 h [30].

Thermocycling was performed 10,000 times, corresponding to a 1-year aging process. The lowest and highest temperatures were set at 5 and 55 °C, respectively. The dwell time and transfer time were set as 28 and 2 s, respectively [31]. Among the 100 specimens, only 50 were subjected to the aforementioned aging process (the remaining samples were excluded from this aging process).

Shear bond testing was performed with a universal testing machine (Instron 5942; Instron Corp., Norwood, MA, USA) at a speed of 1 mm/min until the adhesion of the specimens failed; this adhesion failure was monitored using the testing machine software (Bluehill 2 software, Instron Corp., Norwood, MA, USA). The bond strength (MPa) of each specimen was calculated by dividing the peak load (in N) by the surface area (12.56 mm^2^) [32].

All the groups were analyzed using X-ray diffraction (XRD). Phase transformation analysis was performed using an automated X-ray diffractometer (Ultima IV; Rigaku, Tokyo, Japan) emitting Cu Kα radiation at 30 mA and 40 kV. Phase identification was accomplished using a search-match software (JADE 9; Materials Data, Inc., Livermore, CA, USA), the data for which were provided by the International Centre for Diffraction Data (ICDD, Newtown Square, PA, USA) [33].

In addition, one representative specimen from each group was subjected to surface roughness analysis. The roughness was measured at five points on each specimen with a three-dimensional (3D) optical profiler (Contour GT; Bruker, Billerica, MA, USA). Vision 64 software was used to calculate the surface roughness (*R*_a_) and was implemented three-dimensionally.

In addition, surface characterization of the etched zirconia surfaces was performed with field-emission scanning electron microscopy (SEM; JEOL-7800F; JEOL Ltd., Tokyo, Japan). The surface-treated and control-group specimens were cleaned using a steam cleaner and an ultrasonic cleaner before being dried. The surface microstructures of the specimens were then observed and captured at different magnifications.

The normality test result showed a normal distribution. Statistical analysis was performed using a statistical analysis software program (SPSS version 22 SPSS Statistics, IBM, Armonk, NY, USA). The paired *t*-test was used to determine the groups with a significant difference in data before and after thermocycling. The differences among the mean results for the groups before thermocycling (N-P, N-S, N-Z, N-M, and N-C) and those for the groups after thermocycling (T-P, T-S, T-Z, T-M, and T-C) were subjected to a one-way analysis of variance (ANOVA). Finally, the data were subjected to a two-way ANOVA and the post hoc Tukey test to simultaneously analyze the effects of two factors: the type of surface treatment and whether aging was performed (α = 0.05 for all tests) [30].

## 3. Results

The mean shear bond strengths of the specimens and their standard deviations are presented in Table 3.

By comparing the shear bond strengths of each surface-treated group before and after thermocycling (such as N-P and T-P) using the paired *t*-test, it was found that the shear bond strengths of all the groups differed significantly before and after thermocycling (*p* < 0.001). Analyzing the differences in shear bond strength before thermocycling through a one-way ANOVA revealed that the differences between groups N-C, N-M, N-P, N-S, and N-Z were all significant (*p* < 0.001). Meanwhile, post hoc analysis showed that groups N-S and N-Z had statistically significantly larger values than the N-C group, whereas groups N-M, N-S, and N-Z had statistically significantly larger values than the N-P group. Analyzing the differences in shear bond strength after thermocycling through a one-way ANOVA showed that the differences between groups T-C, T-M, T-P, T-S, and T-Z were all significant (*p* < 0.001). Post hoc analysis showed that the T-M group had statistically significantly larger values than groups T-C, T-P, T-S, and T-Z. The results of a two-way ANOVA indicated that there were statistically significant interactions between the bond strength and surface treatment of the zirconia surface (*p* < 0.001). Additionally, statistical analysis using a two-way ANOVA suggested that the bond strength varied according to whether thermocycling was performed (*p* < 0.001).

Among the groups that did not undergo thermocycling, the bond strengths of the N-S, N-M, and N-Z groups were significantly higher than that of the control group (N-P), whereas the N-C group did not exhibit a statistically significant difference. Furthermore, the bond strength of the N-M group was similar to but slightly lower than that of the N-S group, whereas that of the N-Z group was higher than the latter. However, neither group showed a statistically significant difference from the N-S group (*p* > 0.05). Therefore, an effective increase in bond strength compared to that attained via sandblasting was not observed for the samples treated using the commercial surface-etching systems.

**Figure 1:**
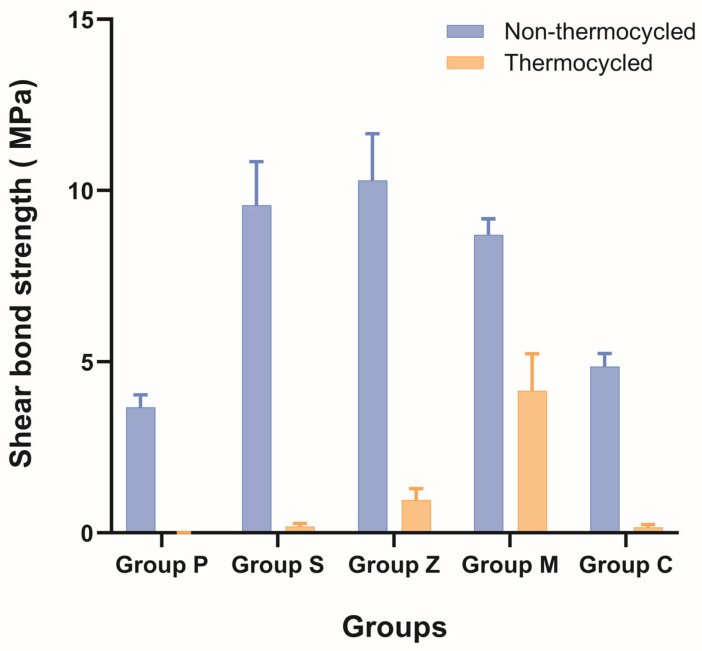
Shear bond strengths (MPa) of different surface treatment groups (*n* = 10 per subgroup). Data are expressed as mean and standard error values.

Upon completing artificial aging, all the groups exhibited a statistically significant decrease in the bond strength. Groups S, P, Z, and C showed large decreases in the bond strength and adhesion failure rate after aging. However, group M exhibited an adhesion failure rate of zero and only a small reduction in bond strength compared with the other groups (Figure 1).

**Figure 2:**
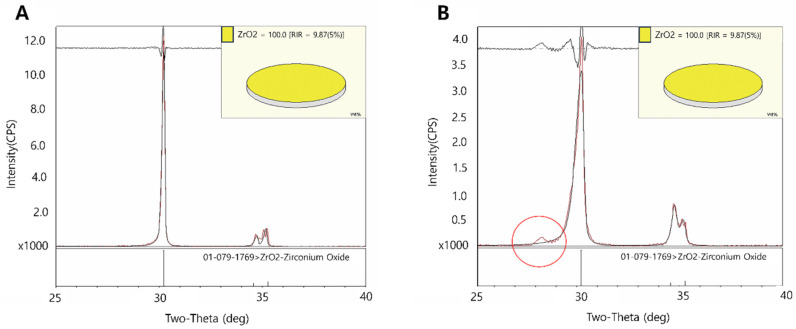

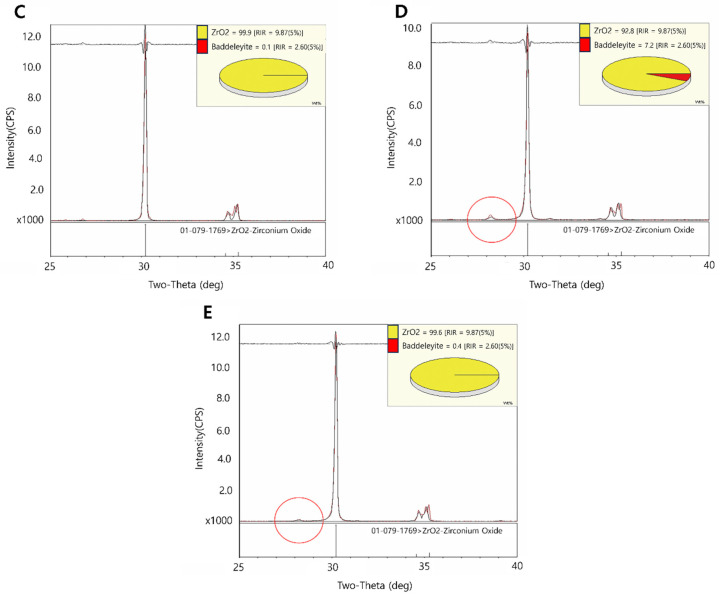
Representative XRD patterns of non-aged groups: (**A**) N-P, (**B**) N-S, (**C**) N-Z, (**D**) N-M, and (**E**) N-C. The red circles indicate the peaks of monoclinic zirconia structures.

The representative XRD patterns obtained from the five groups are presented in Figure 2. The tetragonal phase structure is the main structure in modern zirconia. However, a monoclinic phase structure was detected in the N-S, N-M, and N-C groups. In contrast, the representative peak of the monoclinic phase was not observed in the XRD patterns of the N-P and N-Z groups.

**Figure 3:**
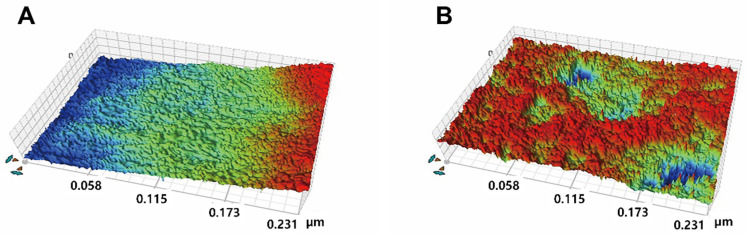

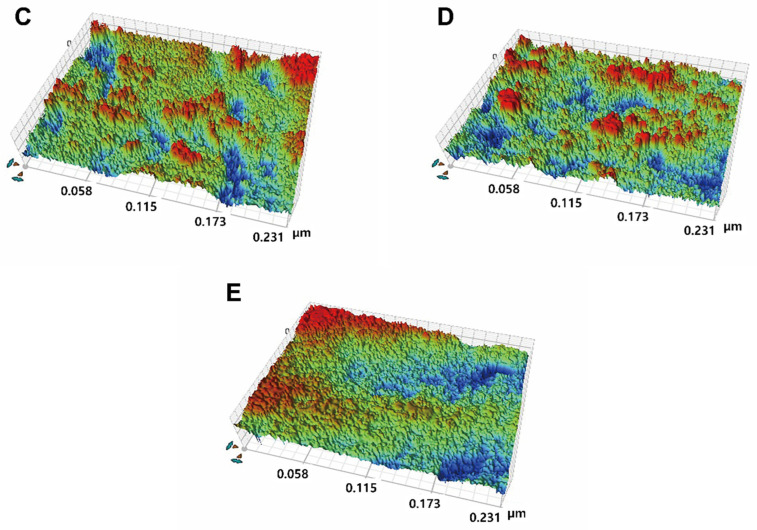
Representative roughness images of non-aged groups: (**A**) N-P, (**B**) N-S, (**C**) N-Z, (**D**) N-M, and (**E**) N-C.

A 3D image of the surface of each zirconia group is shown in Figure 3. The surface curvature of the N-S group was larger than that of the other groups; this result supports the high *R*_a_ value of the N-S group.

**Figure 4:**
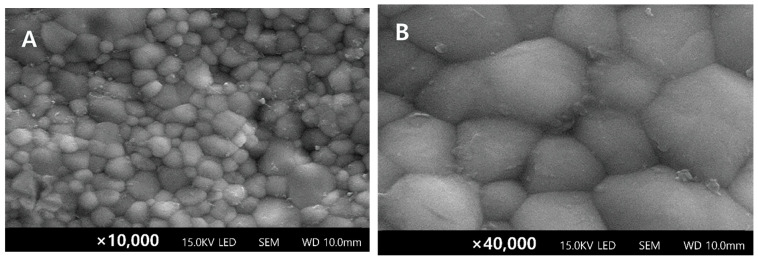

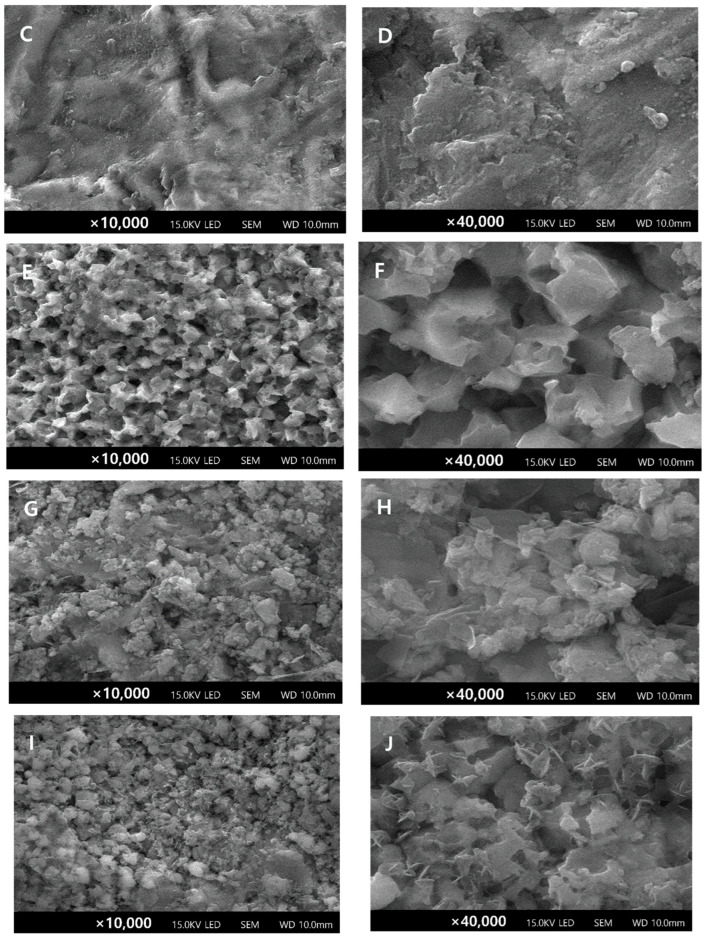
Representative SEM images of surface-treated sites of zirconia specimens at two magnifications (×10,000 and ×40,000): (**A**,**B**) N-P, (**C**,**D**) N-S, (**E**,**F**) N-Z, (**G**,**H**) N-M, and (**I**,**J**) N-C.

SEM images of the surface-treated zirconia specimens at 2× magnification are shown in Figure 4. For the N-S and N-Z groups, which exhibited high bond strength, undercuts were observed on the surface; their presence resulted in a structure that could mechanically interlock with cement.

## 4. Discussion

From the results of this study, the null hypothesis that the pretreatment of zirconia with a commercial etching agent leads to a higher adhesion strength than air abrasion alone was rejected. Sandblasting pretreatment before zirconia cementation resulted in a higher shear bond strength [34], and the strong-acid etching of zirconia resulted in surface changes that strengthened its shear bond with resin cement [35,36].

However, comparisons between the effects of sandblasting and acid pretreatments have not been discussed in detail. Several etching systems were proposed for the pretreatment of zirconia before the bonding process; they are based on methods such as etching by applying heat [37], using an acid with appropriate acidity, and using a strong acid for an extended period. Zircos-E etching is a strong-acid etching method; thus, it is crucial to set an appropriate etching time. If the etching time is excessive, the adhesive strength may be reduced. Cho et al. reported that the *R*_a_ values of zirconia specimens, as observed from SEM images, were higher after etching for 2 h than after etching for 1 and 3 h [38]. Therefore, an etching time of 2 h, as recommended by the manufacturer, is appropriate.

In the case of an experiment using a universal test machine of the current experiment design, the bond strength is measured by the force pushing one point; thus, it is difficult to state that it is a complete measurement of bond strength with a uniform force applied throughout. Additionally, there are limitations in experiment design because it is difficult to separate the material specimen and the adhesive specimen with the force applied uniformly. We believe that the inability to reproduce the forces applied from various directions during mastication in the oral cavity is the main limitation of the study. We assume that if forces are applied from various directions, the bond strength may be lower.

The shear bond test results suggested that the bond strengths of the sandblasted, smart-etched, and Zircos-E-treated specimens were significantly increased compared to those of the control-group specimens; this result agrees well with that of a previous post-zirconia shear bond test study [38]. However, upon acid etching with the cloud system, there was no statistically significant increase in bond strength compared to that of the control group because the cloud system heated the zirconia in the presence of a weak acid and water vapor, causing insufficient modifications to the surface structure of zirconia.

The most commonly used method for artificial aging is thermocycling [22]. However, estimating the number of cycles that correspond to one year of physiological aging in the oral cavity is challenging [39]. Therefore, clear criteria for the number of thermal cycles required are yet to be defined. Gale and Darvell postulated that approximately 10,000 thermal cycles correspond to one year of clinical function, even though most authors have applied fewer cycles [39]. To ensure at least one year of aging, the highest number of thermal cycles, i.e., 10,000, was applied in this study. Only 50 of the 100 specimens (half) were subjected to thermocycling. The specimens were divided thus to compare the simple bond strength without aging and to confirm the stability of the bond strength by checking the change in bond strength after aging.

A comparison between the bond strengths before and after thermocycling revealed that the S and Z groups (group P) exhibited the largest reduction in bond strength after aging. The bond strength of group M (i.e., comparing N-M with T-M) decreased to a lesser extent (group T-M showed the highest bond strength after thermocycling). Therefore, the bond strength of the aged smart-etched specimens was more stable than that of the other groups. We assume that increasing the roughness of the fine surfaces that could not be reached by the sandblasting particles, removing foreign substances, and increasing surface energy resulted in the observed stability of the bond strength with smart etching over time.

From the XRD results, the monoclinic phase structure was detected in the N-S, N-M, and N-C groups. However, no monoclinic phase was observed in the N-Z group. This result suggests that either air abrasion or heat treatment induced the phase change of zirconia. In contrast, the phase change of zirconia did not occur when the surface was treated with the Zircos-E etchant—a strong acid. Moreover, in this study, only the particle transformation from the tetragonal phase to the monoclinic phase was examined through trend analysis of the XRD graph; spectroscopic characterization was not performed through surface analysis via ATR-FTIR or Raman spectroscopy.

The highest average *R*_a_ was obtained for the N-S group. Zircos-E and smart etching resulted in a minimal increase in surface roughness compared to that of the specimens of the N-P group. The *R*_a_ value of the specimens decreased after treatment using the cloud etching system. These results confirmed that commercially available etchants did not significantly increase the average *R*_a_ or form irregularities on the surfaces of the specimens (Figure 3).

The SEM results suggest that the surface roughness increased in all the groups that were subjected to pretreatment before bonding. The N-S (Figure 4B) and N-Z (Figure 4C) groups exhibited the largest increases in surface roughness, and numerous undercuts formed on the surfaces of these groups. The observed high bond strength can be attributed to the mechanical interlocking between the resin cement and the surface of zirconia.

In this study, the effects of various commercial etching solutions on the bond strength of zirconia were investigated. After surface treatment of zirconia, a high bond strength was observed for the N-S and N-Z groups, indicating that sandblasting and strong-acid treatment are effective for increasing the surface roughness of zirconia. However, the bond strength could not be accurately measured because the various forces that are applied in the oral cavity were not considered, and the number of specimens was small. Considering these factors in a follow-up study will help obtain more reliable experimental results.

## 5. Conclusions

In this study, a shear bond test was conducted to determine whether surface pretreatment of zirconia using various acid-etching methods was more effective for increasing the bond strength with resin cement than simple sandblasting. Our results indicated that the bond strength realized after pretreatment using a commercial etching solution before zirconia bonding was not significantly higher than that attained with sandblasting. However, after smart etching, the bond strength after aging was relatively stable compared to those of the other groups. Zircos-E etching did not cause a phase transformation of zirconia and, therefore, resulted in a high bond strength. This study had limitations in its experimental design because it was difficult to separate the material specimen and the adhesive specimen with a uniformly applied force. Additionally, the inability to reproduce forces applied from various directions during mastication in the oral cavity is the main limitation of the study. We assume that forces applied from various directions result in a decreased bond strength. Although further studies that consider factors such as the application of forces in the oral cavity or employ ingenious designs to reproduce forces in various directions to better simulate the oral environment are required for more accurate and detailed results, the findings of this study are expected to aid in developing pretreatment methods that can result in more stable and strong zirconia binding for aesthetic dental restoration.

## Figures and Tables

**Table 1:** Classification of groups based on types of surface treatments for yttria-stabilized tetragonal zirconia blocks.

Group Names	Surface Treatment
N-P, T-P	As-sintered
N-S, T-S	Sandblasting for 15 s with Al_2_O_3_ particles (~50–100 μm, 4.83 bar, 1 cm apart)
N-Z, T-Z	Acid-etched for 2 h with Zircos-E etching solution
N-M, T-M	Sandblasted for 15 s and then acid-etched for 10 min in a hot water bath (80 °C)
N-C, T-C	Acid-etched for 10 min with steam

**Table 2:** Properties of the materials used in this study.

Material	Product Name (Manufacturer)	Content
Zirconia block	Plus Zir Block (DMAX Co.)	ZrO_2_, Y_2_O_3_, H_2_O, Al_2_O_3_
Dual-cured resin cement	Panavia F2.0 (Kuraray)	10-Methacryloxydecyl dihydrogenphosphate photoinitiator Bis-phenol A polyethoxy dimethacrylate
Zirconia etchant	Smart etching (YesBio)	HF, H_2_SO_4_, H_2_O_2_
	Zircos-E (Bioden)	HF, HCl, H_2_SO_4_, HNO_3_, H_3_PO_4_
	Cloud system (MEDIFIVE)	2-Hydroxyethyl methacrylate 10-Methacryloxydecyl dihydrogen phosphate Hydrofluoric acid

**Table 3:** Shear bond strengths (MPa) of different surface treatment groups (Group P: as-sintered; Group S: sandblasted; Group Z: zircos-E; Group M: smart etching; Group C: cloud system) (*n* = 10 per subgroup); data are expressed as mean values.

	Shear Bond Strength (MPa)	
	No Aging Procedure	Aging Procedure
Group P	3.66 ± 1.18	0.01 ± 0.02
Group S	9.57 ± 4.02	0.19 ± 0.28
Group Z	10.29 ± 4.35	0.96 ± 1.05
Group M	8.71 ± 1.46	4.15 ± 3.43
Group C	4.86 ± 1.20	0.16 ± 0.27

## Data Availability

The data presented in this study are available upon reasonable request from the corresponding author.
